# Derepression of Mineral Phosphate Solubilization Phenotype by Insertional Inactivation of *iclR* in *Klebsiella pneumoniae*

**DOI:** 10.1371/journal.pone.0138235

**Published:** 2015-09-18

**Authors:** Mahendrapal Singh Rajput, Bhagya Iyer, Maharshi Pandya, Rahul Jog, Naresh Kumar G, Shalini Rajkumar

**Affiliations:** 1 Institute of Science, Nirma University, Ahmedabad, Gujarat, India; 2 Environmental Molecular Biology Laboratory, Division of Biosphere, Faculty of Environmental Earth Science, Hokkaido University, Sapporo, Hokkaido, Japan; 3 Molecular Microbial Biochemistry Laboratory, Department of Biochemistry, Faculty of Science, Maharaja Sayajirao University of Baroda, Vadodara, Gujarat, India; University of Freiburg, GERMANY

## Abstract

The mode of succinate mediated repression of mineral phosphate solubilization and the role of repressor in suppressing phosphate solubilization phenotype of two free-living nitrogen fixing *Klebsiella pneumoniae* strains was studied. Organic acid mediated mineral phosphate solubilization phenotype of oxalic acid producing *Klebsiella pneumoniae* SM6 and SM11 were transcriptionally repressed by IclR in presence of succinate as carbon source. Oxalic acid production and expression of genes of the glyoxylate shunt (*aceBAK*) was found only in glucose but not in succinate- and glucose+succinate-grown cells. IclR, repressor of *aceBAK* operon, was inactivated using an allelic exchange system resulting in derepressed mineral phosphate solubilization phenotype through constitutive expression of the glyoxylate shunt. Insertional inactivation of *iclR* resulted in increased activity of the glyoxylate shunt enzymes even in succinate-grown cells. An augmented phosphate solubilization up to 54 and 59% soluble phosphate release was attained in glucose+succinate-grown SM6Δ and SM11Δ strains respectively, compared to glucose-grown cells, whereas phosphate solubilization was absent or negligible in wildtype cells grown in glucose+succinate. Both wildtype and *iclR* deletion strains showed similar indole-3-acetic acid production. Wheat seeds inoculated with wildtype SM6 and SM11 improved both root and shoot length by 1.2 fold. However, *iclR* deletion SM6Δ and SM11Δ strains increased root and shoot length by 1.5 and 1.4 folds, respectively, compared to uninoculated controls. The repressor inactivated phosphate solubilizers better served the purpose of constitutive phosphate solubilization in pot experiments, where presence of other carbon sources (e.g., succinate) might repress mineral phosphate solubilization phenotype of wildtype strains.

## Introduction

Mineral phosphate solubilization (MPS) is one of the most desirable traits of plant growth promoting rhizobacteria (PGPR). *Klebsiella* is known for plant growth promoting (PGP) activities like phosphate (P) solubilization, indole-3-acetic acid (IAA) production, siderophore production and HCN production [1–3]. The existence of *Klebsiella pneumoniae* associated to nitrogen fixation in wheat (*Triticum aestivum L*.*)* has been reported [4], while *Klebsiella* strains endophytic to rice, maize, sugarcane and banana have also been reported [5–8].

The principal mode of MPS is organic acid secretion by rhizospheric microorganisms [9]. Among organic acids responsible for MPS phenotype, gluconic acid, 2-ketogluconic acid, oxalic acid, acetic acid, citric acid, succinic acid and glyoxylic acid are the major ones [10]. Organic acid production by bacteria depends on the carbon source utilized, as the organic acids produced are intermediates of the central carbon metabolism.

The central metabolic pathways in bacteria are controlled by a set of global and local regulators depending on the carbon sources available and the culture conditions [11]. Carbon catabolite repression (CCR) controls the uptake and metabolism of a preferred carbon source when bacteria are exposed to more than one carbon sources. CCR has been extensively studied in the *Enterobacteriaceae* and *Firmicutes* where it is mediated by components of the phosphoenolpyruvate (PEP): carbohydrate phosphotransferase system (PTS), but the mechanisms are different in these groups [12]. In *E*. *coli*, the preferential utilization of glucose over other carbon sources is controlled by catabolite repression protein (CRP) and PTS [13].

In our previous report, we have shown that *Klebsiella pneumoniae* SM6 and SM11 solubilized mineral phosphate by production of oxalic acid when grown in glucose as sole carbon source [14]. However, acid production and MPS phenotype of both these strains were repressed in the presence of succinate [14]. Oxalic acid was produced in these bacteria from glyoxylate by the action of glyoxylate oxidase (GO) [14]. Genes of the glyoxylate shunt in the *Enterobacteriaceae* family are clustered in the *aceBAK* operon under the control of an isocitrate lyase repressor (IclR) [15, 16]. The *aceBAK* operon codes for malate synthase (*aceB*), isocitrate lyase (*aceA*) and isocitrate dehydrogenase kinase/phosphatase (*aceK*) [15, 16]. Isocitrate lyase (ICL) cleaves isocitrate into glyoxylate and succinate, malate synthase (MS) catalyzes the condensation of acetyl-CoA and glyoxylate to form malate, while isocitrate dehydrogenase kinase/phosphatase inactivates/activates isocitrate dehydrogenase [15, 16].

The IclR class of transcriptional regulators was first reported in *E*. *coli* [17, 18]. Regulators of the IclR family have been shown to control diverse cell functions like carbon metabolism in the *Enterobacteriaceae* [19], catabolism of aromatic compounds in soil bacteria [20], quorum-sensing signals in *Agrobacterium* [21], and plant virulence in certain enterobacteria [22]. IclR (glyoxylate shunt repressor) is the best-characterized member of the IclR family in *E*. *coli*, which regulates the *aceBAK* operon encoding proteins for acetate utilization [15, 16].

In the present study, we investigated the role of *iclR* in catabolite repression of oxalic acid production in P solubilizing *Klebsiella pneumoniae* SM6 and SM11. To understand the molecular mechanism of succinate mediated repression of oxalic acid linked MPS phenotype, *ΔiclR* mutants *Klebsiella pneumoniae* SM6Δ and SM11Δ were generated and assessed for relieved repression of MPS phenotype. Although both strains are genetically similar and produce oxalic acid, they vary considerably in quantity of oxalic acid produced and activities of ICL and GO, as described previously [14]. Hence, both strains were considered for further studies. Based on expression profile, enzyme activities and physiological studies, we have identified *iclR* as putative repressor of oxalic acid linked MPS phenotype in our isolates. *iclR* inactivation showed derepression of the glyoxylate shunt enzymes and enhanced the MPS phenotype in presence of succinate and glucose. To check the effect of wildtype and *ΔiclR* strains on plant growth, pot experiments were conducted under controlled conditions. Indeed, the deletion of *iclR* improved plant growth, compared to the wildtype.

## Materials and Methods

### Bacterial strains, plasmids and growth conditions

The bacterial strains and plasmids used in this study are listed in Table 1. *E*. *coli* DH5α, *K*. *pneumoniae* SM6, *K*. *pneumoniae* SM11 *K*. *pneumoniae* SM6Δ and *K*. *pneumoniae* SM11Δ were grown aerobically on LB medium (Himedia Laboratories, India) at 30°C. *K*. *pneumoniae* SM6 and *K*. *pneumoniae* SM11 were deposited at Microbial Culture Collection (MCC), Pune with accession numbers MCC2730 and MCC2716, respectively. For plate cultures, 1.5% agar was added to LB medium before autoclaving. Temperature sensitive mutants and temperature sensitive plasmid-carrying strains were grown at 28°C. Antibiotics ampicillin, erythromycin and kanamycin were added to the medium at a concentration of 50 μg ml^-1^, as and when required. Culture stocks were preserved in 50% (v/v) glycerol and stored at -20°C. Antibiotics, salts, reagents and carbon sources were acquired from Merck-Millipore, Germany.

**Table 1:** Bacterial strains and plasmids used in the study.

Strains	Relevant Characteristics	Source or Reference
*E*. *coli* DH5α	Host, *ΔlacU169 (ϕ80lacZΔM15) recA1*	[23]
*K*. *pneumoniae* SM6	P solubilizing, free-living N_2_ fixer, Host, Wildtype (GenBank accession number JX139003)	[14]
*K*. *pneumoniae* SM11	P solubilizing, free-living N_2_ fixer, Host, Wildtype (GenBank accession number JX139004)	[14]
*K*. *pneumoniae* SM6Δ	*ΔiclR*, P solubilizing, free-living N_2_ fixer, Host	This study
*K*. *pneumoniae* SM11Δ	*ΔiclR*, P solubilizing, free-living N_2_ fixer, Host	This study
**Plasmids**		
pUC18	Amp^R^, *LacZα*, pBR322 origin, cloning vector	[24]
pcDNA3.1 (+)	Kan^R^, Amp^R^, T7 promoter, CMV promoter, Mammalian expression vector	Invitrogen, USA
pGRG36	Amp^R^, Tn7 insertion vector, pSC101 origin of replication *oriTS*	[25]
pRV85	Ery^R^, *gfp*_uv_, *pldh*L promoter	[26]
pSMB1a	386 bp SM6 *iclR*-N’ fragment at PstI:XbaI site of pUC18, Amp^R^, pBR322 origin	This study
pSMB2a	363 bp SM6 *iclR*-C’ fragment at SmaI:SacI site of pSMB1a, Amp^R^, pBR322 origin	This study
pSMB3a	890 bp *kanR* gene at BamHI site of pSMB2a, Amp^R^ pBR322 origin,	This study
pSMB4a	pSC101 temperature sensitive origin of replication, cloned in NdeI:PciI digested pSMB3a, *oriTS*, *iclR*-N’, *iclR*-C’, Kan^R^	This study
pSMB5a	*pldhL*:*gfp*_*uv*_ fragment from pRV85 digested with EcoRI, cloned at EcoRI site of pSMB4a, pSC101 *oriTS*, *iclR*-N’, *iclR*-C’, Kan^R^	This study
pSMB1b	386 bp SM11 *iclR*-N’ fragment at PstI:XbaI site of pUC18, Amp^R^, pBR322 origin	This study
pSMB2b	363 bp SM11 *iclR*-C’ fragment at SmaI:SacI site of pSMB1b, Amp^R^, pBR322 origin	This study
pSMB3b	890 bp *kanR* gene at BamHI site of pSMB2b, Amp^R^ pBR322 origin,	This study
pSMB4b	pSC101 temperature sensitive origin of replication, cloned in NdeI:PciI digested pSMB3b, *oriTS*, *iclR*-N’, *iclR*-C’, Kan^R^	This study
pSMB5b	*pldhL*:*gfp*_*uv*_ fragment from pRV85 digested with EcoRI, cloned at EcoRI site of pSMB4b, pSC101 *oriTS*, *iclR*-N’, *iclR*-C’, Kan^R^	This study

Amp^R^ ampicillin resistance, Kan^R^ kanamycin resistance, Ery^R^ erythromycin resistance, *oriTS* temperature sensitive origin of replication, *pldhL Lactobacillus* lactate dehydrogenase promoter, *gfp*_*uv*_ UV excitable green fluorescent protein.

### Growth studies and enzyme assays

Effect of carbon source on growth of wildtype and mutant strains was studied as described previously [14]. To study mono- and diauxic growth of the cultures, they were grown in M9 minimal medium with 20 mM glucose, 20 mM succinate and 10 mM glucose+succinate as carbon source. Glucose utilization in liquid media was quantified by Glucose-SLR Reagent (Lab-Care Diagnostics, India) [14]. Activities of ICL and GO were determined in cell extracts of glucose-, succinate- and glucose+succinate-grown cells as described previously by Dixon and Kornberg [27] and Akamatsu and Shimada [28], respectively, with some modifications [14]. Cell extract was prepared as described by Buch et al. [29], with mid-log phase cells, harvested by centrifugation at 12,000 rpm for 5 min at 4°C. The cell pellet was washed with 80 mM phosphate buffer (pH 7.5) and resuspended in small volume of same buffer with 20% glycerol and 1 mM DTT. The cell suspension was sonicated using Ultrasonic homogenizer JY92-IIDN (Syclon, China) for 2 min at a pulse rate of 30 at 500 Hz on ice. Cell debris were removed by centrifugation at 12,000 rpm at 4°C for 30 min and the supernatant was used for enzyme assays.

### Determination of MPS phenotype

Qualitative and quantitative estimation of phosphate solubilization was carried out to assess if *iclR* inactivation relieved succinate-mediated repression of MPS phenotype in SM6Δ and SM11Δ strains. Qualitative estimation of MPS phenotype of wildtype and mutant strains was performed on Pikovskaya agar [30] and Tris rock phosphate (TRP) agar, as described previously [14]. TRP agar contained 100 mM glucose, 100 mM succinate or equimolar mixture of glucose and succinate (50 mM each; repression medium) as carbon sources; Pikovskaya agar contained glucose (10 g L^-1^) or equimolar mixture of glucose and succinate (5 g L^-1^ each; repression medium) as carbon sources; Pikovskaya broth contained glucose (10 g L^-1^) or equimolar mixture of glucose and succinate (5 g L^-1^ each; repression medium) as carbon sources. Quantitative estimation of P solubilization was carried out in Pikovskaya broth, as described by Rajput et al [14], and phosphate released was quantified as described by Ames [31]. In this method, soluble phosphate reacts with ammonium molybdate to give phosphomolybdate, which is further reduced by ascorbic acid to give a blue colored complex that can be quantified at 820 nm [31].

### Polymerase chain reaction (PCR) and product purification

PCR amplifications were carried out for expression studies and vector construction. Primers used for DNA amplification are listed in Table 2. Primers for *K*. *pneumoniae* SM6, SM11, SM6Δ and SM11Δ were designed using nucleotide sequences of closely related *K*. *pneumoniae* strains available at NCBI. Genomic DNA was isolated from overnight grown cultures using GeneJET genomic DNA purification kit (Thermo Scientific, USA) following the manufacturer’s instructions. Plasmid isolation from cells grown in LB medium with ampicillin, erythromycin and kanamycin was performed using a GeneJET plasmid miniprep kit (Thermo Scientific, USA). pcDNA3.1 was used as PCR template for amplification of kanamycin resistance gene, pGRG36 for pSC101 *oriTS*, and genomic DNA of *K*. *pneumoniae* SM6 and SM11 for *iclR*-N’ and *iclR*-C’ fragments. All PCR amplifications were carried out in a Nexus Gradient Mastercycler (Eppendorf, Germany). The reaction mixture for PCR amplification was as follows: 200 ng template DNA, 200 μM of each of the four dNTPs, 20 pmol of each forward and reverse primer, 2 U Maxima Hot Start *Taq* DNA polymerase (Thermo Scientific, USA) and 1X Maxima Hot Start *Taq* Buffer (Thermo Scientific, USA), in final volume of 20 μl. The PCR parameters were as follows: 4 min initial denaturation at 95°C, 35 X (1 min denaturation at 95°C, 1 min primer annealing at 50–60°C (depending on the Tm and G+C content of the primers, see Table 2), 0.5–2.5 min primer extension at 72°C (see Table 2)) and a final extension of 10 min at 72°C. PCR products were analysed on 1% agarose gel. PCR products used for cloning were purified using GeneJET gel extraction and DNA cleanup micro kit (Thermo Scientific, USA). Primers were procured from Integrated DNA Technologies, Inc. (Coralville, USA)

**Table 2:** Primers used in the study.

Primers	Relevant Characteristics (5’→3’)	Annealing Temperature	Primer Extension time
IclRN’-F	AATCTGCAGTTCGCGATGGCCACTAC, with PstI underlined site, located 58 bp downstream of *iclR* start codon	55°C	0.5 min
IclRN’-R	CCGTCTAGAGAGCACCGCCAGATTCA, with XbaI underlined site, located 445 bp downstream of *iclR* start codon	55°C	0.5 min
IclRC’-F	TATCCCGGGGACCACCAGGCGATCATTA, with SmaI underlined site, located 454 bp downstream of *iclR* start codon	58°C	0.5 min
IclRC’-R	ACGGAGCTCCATCGGTCATACGCGAGAT, with SacI underlined site, located 818 bp downstream of *iclR* start codon	58°C	0.5 min
KanR-F	CCCGGATCCCCGGGAGCTTGTATATCCATTT, with BamHI underlined site, located 60bp upstream of *kanR* start codon	57°C	1 min
KanR-R	CCCGGATCCTCGCTTGGTCGGTCATTTC, with BamHI underlined site, located 17 bp downstream of *kanR* stop codon	57°C	1 min
OriTS-F	CCCACATGTCCCTGTAGCGGCGCATTAAG, with PciI underlined site, located 973 bp upstream of pSC101 *oriTS*.	59°C	2.5 min
OriTS-R	AAACATATGTGGTGAACAGCTTTAAATGCACC, with NdeI underlined site, located 1098 bp downstream of pSC101 *oriTS*.	59°C	2.5 min
AceB-F	CACCGATGAACTGGCCTTTA, binds 21 bp downstream of *aceB* start codon	54°C	0.5 min
AceB-R	GATTGGGCTGCAACTGATAGA, binds 478 bp downstream of *aceB* start codon	54°C	0.5 min
AceA-F	CCGCGCTTTATCGACTACTT, binds 433 bp downstream of *aceA* start codon	54°C	0.5 min
AceA-R	AGATCCGGTTTCGACGTTTC binds 865 bp downstream of *aceA* start codon	54°C	0.5 min
AceK-F	GTAAACGCCTTATCCGGTCTAC, binds 35 bp downstream of *aceK* start codon	54°C	0.5 min
AceK-R	CGTGATGCGGTAGAAGAGTATG, binds 462 bp downstream of *aceK* start codon	54°C	0.5 min
IclR-F	TCATCGGTCATACGCGAGAT binds 77 bp downstream of *iclR* start codon	54°C	0.5 min
IclR-R	CGCCTGCTGATGGAAGATT binds 480 bp downstream of *iclR* start codon	54°C	0.5 min
GyrA-F	GGAAATCAGGGCCAGGAATATG, binds 1983 bp downstream of gyrA start codon	54°C	0.5 min
GyrA-R	CGTTGGTGACGTAATCGGTAAA, binds 2407 bp downstream of *gyrA* start codon	54°C	0.5 min

Restriction sites added at the 5’ end of primer are underlined.

### Expression studies

Total RNA was isolated from glucose-, succinate- and glucose+succinate-grown *K*. *pneumoniae* SM6, SM11, SM6Δ and SM11Δ cells using GeneJET RNA purification kit (Thermo Scientific, USA) following manufacturer’s instructions, except that the cells were washed twice with sterile NaCl solution (0.8%) and resuspended in the same solution before RNA purification. Purified RNA was quantified by measuring absorbance at 260 nm, and the quality and purity was confirmed by electrophoresis on 1.5% agarose gel containing 1.9% (v/v) formaldehyde. cDNA was synthesized from isolated RNA using First Strand cDNA synthesis kit (Thermo Scientific, USA) following manufacturer’s instructions. cDNA was used as template for analysing expression of *aceB*, *aceA*, *aceK*, *iclR* and *gyrA* genes in glucose-, succinate- and glucose+succinate-grown cells. DNA Gyrase A (*gyrA*) was used as internal control to standardize expression and mRNA levels.

### Construction of *iclR* mutants by insertion of a kanamycin cassette

The *iclR* gene was inactivated employing an allelic exchange system in SM6 and SM11 strains to generate SM6Δ and SM11Δ strains, respectively. To achieve this, pSMB5a and pSMB5b vectors were constructed with 386 bp and 363 bp *iclR* N’ and C’ gene fragments (of SM6 and SM11, respectively) flanking kanamycin resistance gene, *pldhL*:*gfp*_*uv*_, and pSC101 temperature controlled origin of replication to direct the integration and excision of vector. *E*. *coli* DH5α was used routinely as a host strain for cloning experiments. DNA was introduced into *E*. *coli* using Bacterial TransformAid kit (Thermo Scientific, USA). 386 bp *iclR*-N’ PstI-XbaI fragment was amplified from genomic DNA of *K*. *pneumoniae* SM6 and SM11 using primers IclRN’-F and IclRN’-R. The amplicon was cloned in pUC18 digested with PstI-XbaI to generate pSMB1a and pSMB1b, respectively. 363 bp *iclR-*C’ SmaI-SacI fragment was amplified with IclRC’-F and IclRC’-R primers and cloned in SmaI-SacI digested pSMB1a and pSMB1b, generating pSMB2a and pSMB2b, respectively. Kanamycin resistance gene from pcDNA3.1(+) was amplified using KanR-F and KanR-R primers with BamHI sites. 890 bp *kanR* gene was ligated at *Bam*HI site of pSMB2a and pSMB2b between *iclR-N’* and *iclR-C’* fragments creating pSMB3a and pSMB3b, respectively. Successful insertion was confirmed by kanamycin resistant colonies on LB agar plate with kanamycin (50 μg ml^-1^), restriction digestion of isolated plasmids and amplification of kanamycin resistance gene from pSMB3a and pSMB3b. 2297 bp *oriTS* was amplified from pGRG36 using oriTS-F and oriTS-R primers with PciI and NdeI flanking sites. pSMB3a and pSMB3b were then digested with PciI and NdeI, and resultant 2288 bp fragment was ligated with 2297 bp PciI and NdeI digested *oriTS*. Thus, ampicillin resistance gene with pBR322 origin of replication in pSMB3a and pSMB3b was replaced by pSC101 temperature sensitive origin of replication to give pSMB4a and pSMB4b. pRV85 was digested with EcoRI to release *pldhL*:*gfp*_*uv*_ fragment and was cloned at EcoRI site of pSMB4a and pSMB4b. Restriction enzymes were acquired from New England Biolabs (Ipswich, Mass.). S1 Fig shows diagrammatic representation of pSMB5a and pSMB5b construction. pSMB5a-transformed SM6 and pSMB5b-transformed SM11 cells were grow on LB medium with kanamycin and checked for fluorescence under UV light at 28°C (permissive temperature for replication of *oriTS*) and at 40°C (non-permissive temperature) for integration into genome. The *ΔiclR* strains were selected for kanamycin resistance and absence of green fluorescence. The integration of pSMB5a and pSMB5b in the genome of SM6 and SM11 strains, respectively, was verified by PCR, loss of green fluorescence and expression of *iclR*. For PCR verification, chromosomal DNA of transformants was amplified using specific forward primer annealing to regions upstream of *iclR* and reverse primer of kanamycin resistance gene (data not shown). The loss of green fluorescence was due to curing of pSMB5a and pSMB5b. *iclR* inactivation and resultant expression of *aceBAK* operon genes were also confirmed by expression studies.

### Indole-3-acetic acid (IAA) production

*K*. *pneumoniae* wildtype and *ΔiclR* strains were screened for IAA production in the LB medium containing tryptophan (1 mg ml^-1^). The medium was inoculated with 100 μl of overnight-grown bacterial culture and incubated on orbital shaker at 150 rpm for 48 h at 28°C. Quantitative estimation of IAA production was performed, as described by Gordon and Weber [32], using Salkowski reagent containing 10 g L^-1^ (NH_4_)_2_MoO_4_, 10 ml L^-1^ H_2_SO_4_ and 5 g L^-1^ FeSO_4_.7H_2_O. Uninoculated medium was taken as control.

### Pot experiments

Pot experiments were carried out with Wheat (var. Lokwan) (*Triticum aestivum*), as described by Sachdev et al [33]. Soil samples were collected, air dried, sieved (2 mm mesh) and sterilized by repeated cycles of autoclaving. pH and total P content of the soil was determined by AES-ICP (Model: 330RL, Perkin-Elmer). Soil used for pot experiments was brown, sandy and loamy, with pH 7.3 and total P content 0.12 g L^-1^. Approximately 1 kg of sterile soil was distributed per pot, and sterile seeds were inoculated with SM6, SM11, SM6Δ and SM11Δ strains (10^6^−10^7^ cfu ml^-1^) while seeds treated with sterile medium served as control. All pots were maintained in a greenhouse under natural sunlight (10 h day and 14 h night cycle), temperature (average 25°C) and supplied with 150–200 ml autoclaved water per pot per day. Plants were gently uprooted on 31^st^ day and scored for root length (cm), shoot length (cm), root: shoot ratio and dry mass (g).

### Statistical analysis

All the experiments were performed in triplicates, and the values in results are mean ± standard deviation, averaged for data analysis. The plant experiment data were expressed as mean ± standard deviation and an analysis of variance (ANOVA) to test the significance of differences was performed with the statistical program Prism® (GraphPad 5.0, USA). The differences between the parameters were evaluated using Tukey’s test (Prism® software). P values ≤ 0.05 were considered to be statistically significant.

## Results

### Expression analysis of *iclR* and *aceBAK* operon genes

**Fig 1:**
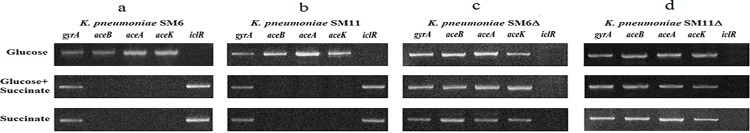
Expression profile of *aceBAK* operon genes and *iclR* in glucose-, glucose+succinate-and succinate-grown *K*. *pneumoniae* strains (a) SM6, (b) SM11, (c) SM6Δ and (d) SM11Δ.

The differential operation of the glyoxylate shunt in glucose-, succinate- and glucose+succinate-grown *K*. *pneumoniae* SM6 and SM11 was studied through expression analysis of the glyoxylate shunt (i.e. *aceBAK* operon) genes and its transcriptional repressor *iclR*. Genes of the glyoxylate shunt *aceB*, *aceA* and *aceK* were expressed only when glucose was the sole carbon source, whereas *iclR* was expressed when succinate was the sole carbon source or when present along with glucose (Fig 1A and 1B).

### *iclR* inactivation and confirmation

*iclR* deletion strains SM6Δ and SM11Δ retained kanamycin resistance while *gfp*_uv_ used as second selectable marker was lost after the second recombination. This was due to plasmid curing of pSMB5a and pSMB5b when transformant SM6 and SM11 were subjected to reinouclation in LB with kanamycin and temperature cycles, and *kanR* integration disrupts *iclR* (S2 Fig). Fig 1C and 1D show expression analysis of *aceBAK* operon genes and *iclR* of glucose-, succinate- and glucose+succinate-grown SM6Δ and SM11Δ. Expression of *iclR* was not observed in succinate- and glucose+succinate-grown *iclR* inactive SM6Δ and SM11Δ.

### Physiological activities and enzyme activities of the glyoxylate shunt and GO

**Fig 2:**
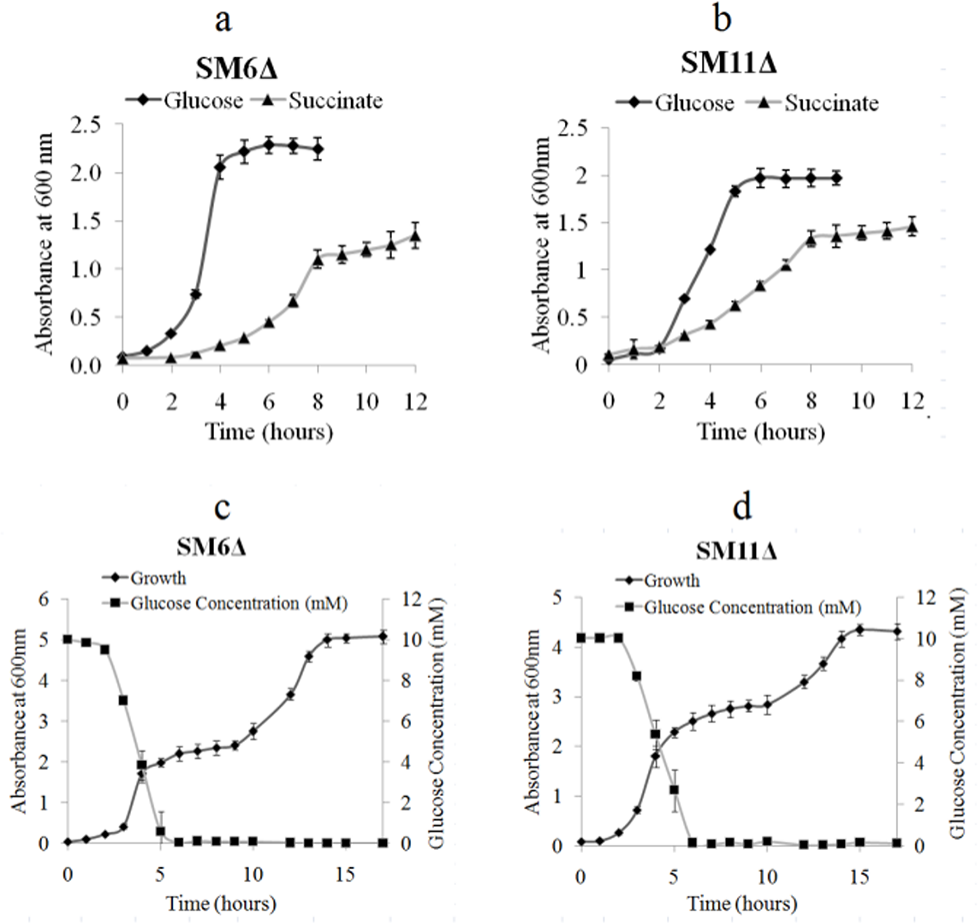
Monoauxic growth profile of (a) SM6Δ and (b) SM11Δ, and diauxic growth profile of (c) SM6Δ and (d) SM11Δ.

**Fig 3:**
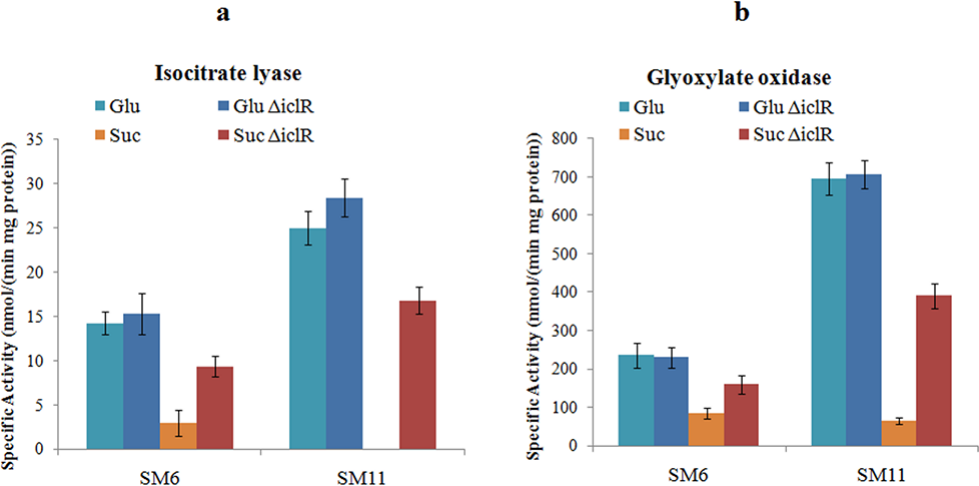
Enzyme activity of glucose- and succinate-grown SM6, SM11, SM6Δ and SM11Δ: (a) ICL (b) GO.

Fig 2A and 2B show monoauxic growth profile of SM6Δ and SM11Δ when grown on glucose and succinate. No significant difference in growth pattern was observed for both mutants in either of the carbon sources as compared to the wildtype cultures. Glucose favoured the maximum growth, and cells entered exponential phase within 2–3 h of inoculation, reaching maximum optical density (OD) with stationary phase in 4–5 h. Growth on succinate was observed after a lag phase and was slower than on glucose. This may be because succinate is a poor carbon source for enterobacteria like *E*. *coli* [34]. Similarly, no significant difference was observed in the diauxic growth profile and carbon utilization pattern of *iclR* mutants SM6Δ and SM11Δ, as compared to wildtype SM6 and SM11. Glucose was utilized preferentially over succinate in both the mutants and 90–100% of glucose was consumed within first 5–6 h of incubation, while succinate was utilized only when glucose was exhausted in the media (Fig 2C and 2D). The specific activities of ICL and GO in glucose- and succinate-grown SM6 and SM11 cells and their *iclR* inactivated counterparts SM6Δ and SM11Δ are depicted in Fig 3.

**Fig 4:**
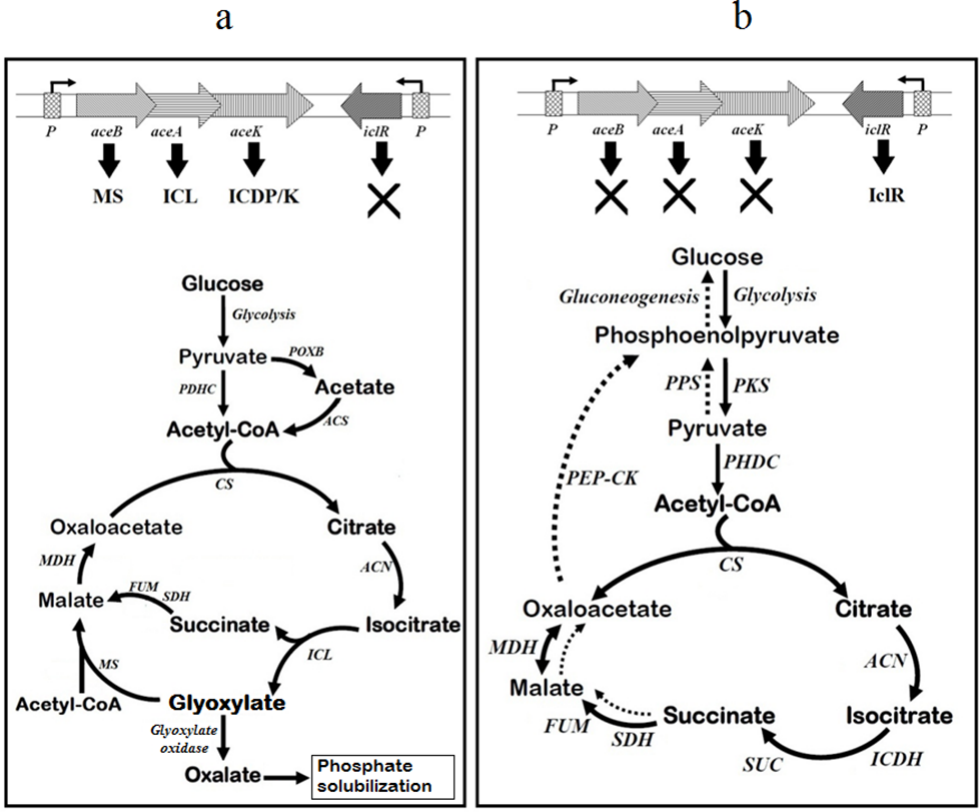
Carbon metabolism linked P solubilization in (A) glucose and (B) succinate or glucose+succinate-grown *Klebsiella pneumoniae* SM6 and SM11. Glucose is metabolized by EMP pathway and acetate is overproduced, which can be then activated to acetyl-CoA. Acetyl-CoA is incorporated in the TCA and the glyoxylate shunt. During growth on succinate, gluconeogenesis is required for the formation of PEP and further intermediates of the EMP pathway, whereas the TCA cycle serves as the main catabolic process under these conditions. MS, malate synthase; ICL, isocitrate lyase; ICDP/K, isocitrate dehydrogenase kinase/phosphatase; IclR, isocitrate lyase repressor; *P*, promoter; POXB, pyruvate oxidase B; ACS, AMP-forming acetyl-CoA synthetase; PDHC, pyruvate dehydrogenase complex; PPS, phosphoenolpyruvate synthase; PKS, pyruvate kinase; PEP-CK, phosphoenolpyruvate carboxykinase; CS, citrate synthase; ACN, aconitase; SUC, succinyl-CoA synthetase complex; ICDH, isocitrate dehydrogenase; SDH, succinate dehydrogenase; FUM, fumarase; MDH, malate dehydrogenase.

*iclR* inactivation resulted in 3.2 fold increase in ICL activity of succinate-grown SM6Δ, as compared to wildtype SM6, and in high activity in succinate-grown SM11Δ, whereas ICL was undetectable in SM11 strain. The ICL activity of succinate-grown SM6Δ and SM11Δ was 74.6% and 73.2% as compared to that of glucose-grown cells. The ICL activity in succinate-grown wildtype SM6 and SM11 cells was 20.7% and 0% respectively, when compared to the glucose-grown cells [14]. Similarly, an enhanced GO activity was observed in succinate-grown SM6Δ and SM11Δ (Fig 3A). Based on the published data [14] and present findings, we propose a metabolic scheme that explains the MPS phenotype in the wildtype SM6 and SM11 strains during grown on glucose or on succinate (or on glucose+succinate) (Fig 4).

### P solubilization by *iclR* mutants

**Fig 5:**
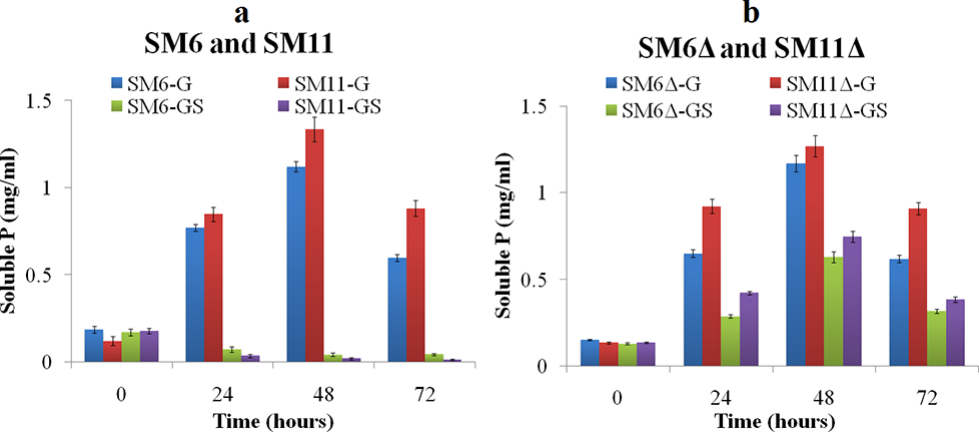
P released by (a) SM6 and SM11, and (b) SM6Δ and SM11Δ in Pikovskaya broth with glucose (G) and glucose+succinate (GS).

**Fig 6:**
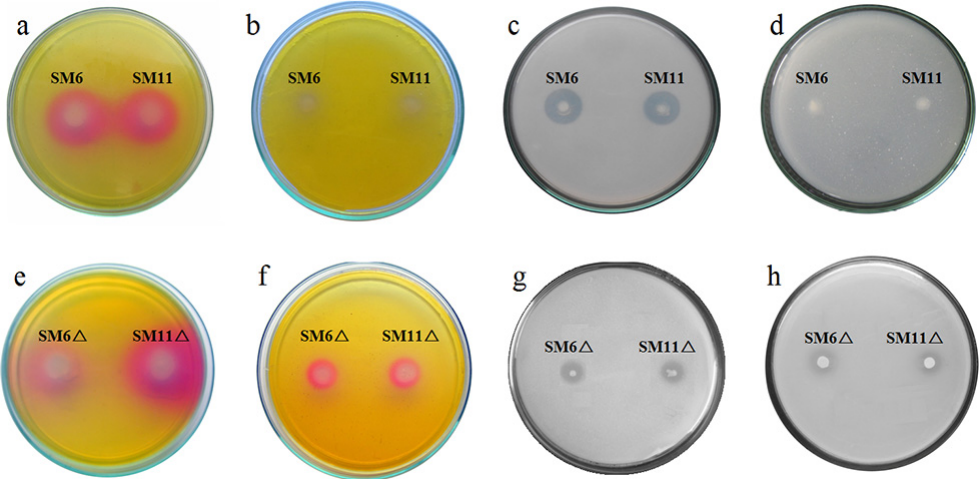
MPS phenotype of wildtype SM6 and SM11 on (a) TRP agar with glucose (b) TRP agar with glucose+succinate (c) Pikovskaya agar with glucose (d) Pikovskaya agar with glucose+succinate, and MPS phenotype of SM6Δ and SM11Δ on (e) TRP agar with glucose (f) TRP agar with glucose+succinate (g) Pikovskaya agar with glucose (h) Pikovskaya agar with glucose+succinate.

Fig 5 shows soluble P released in Pikovskaya broth with glucose and glucose+succinate. The maximum P solubilized by SM6 and SM11 strains in media with glucose+succinate was only 3.8 and 1.5%, respectively, as compared to P solubilized with glucose [14]. The amount of P solubilized in glucose media by the wildtype strains was almost equivalent to that of the corresponding *ΔiclR* strains. SM6Δ and SM11Δ were enhanced in P solubilization in glucose+succinate medium, reaching up to 54 and 59% in comparison to glucose medium. Fig 6 shows mineral phosphate solubilization in Pikovskaya agar and rock phosphate solubilization in TRP agar by SM6, SM11, SM6Δ and SM11Δ in presence of glucose and glucose+succinate.

### IAA Production

SM6 and SM11 produced 18.1 ± 0.9 μg ml^-1^ and 18.7 ± 1.1 μg ml^-1^ of IAA, respectively, while 17.6 ± 1.2 μg ml^-1^ and 19.5 ± 1.1 μg ml^-1^ IAA was produced by SM6Δ and SM11Δ respectively, in LB medium supplemented with tryptophan.

### Effect of wildtype and *iclR* inactivated strains on plant growth

Wildtype and *ΔiclR* strains improved plant growth over the control (Table 3). Plants inoculated with the SM6 and SM11 strains increased root length and shoot length, while the growth improvement with SM6Δ and SM11Δ strains was even more prominent. SM6Δ and SM11Δ treated plants had significantly higher root length and shoot length compared to wildtype.

**Table 3:** Growth parameters of wheat plants in the presence of *K*. *pneumoniae* SM6, SM11, SM6Δ and SM11Δ strains.

	Root length (cm) *	Shoot length (cm) *	Root: Shoot ratio	Dry mass (mg) *
Control	19.2 ± 1.8	16.7 ± 1.7	1.14	241.1 ± 19.5
*K*. *pneumoniae* SM6	25.7 ± 2.16	20.8 ± 1.8	1.23	290.4 ± 17.1
*K*. *pneumoniae* SM11	24.3 ± 1.9	19.6 ± 1.5	1.23	282.9 ± 20.1
*K*. *pneumoniae* SM6Δ	30.2 ± 2.4^†^	23.5 ± 1.7	1.28	324.7 ± 22.8^†^
*K*. *pneumoniae* SM11Δ	29.9 ± 3.1^†^	23.0 ± 1.8^†^	1.3	330.2 ± 24.3^†^

* Significant as compared to control (P ≤ 0.05).

† Significant at P ≤ 0.05 as compared to their respective wildtype.

Values for root length, shoot length and dry mass are mean ± standard deviation of 12 plants (n = 12).

## Discussion

### *iclR* and *aceBAK* expression

Absence of expression of *aceBAK* operon genes was accompanied by the repression of phosphate solubilization and acid production in succinate or glucose+succinate-grown *K*. *pneumoniae* SM6 and SM11 [14]. In *E*. *coli*, IclR regulates the expression of the *aceBAK* operon coding for isocitrate lyase (AceA), malate synthase (AceB) and isocitrate dehydrogenase kinase/phosphatase (AceK) [17]. Isocitrate dehydrogenase kinase/phosphatase (AceK) phosphorylates isocitrate dehydrogenase (ICD), reducing the ICD activity and therefore the carbon flux through tricarboxylic acid (TCA) cycle [35]. Repression of the glyoxylate shunt genes is relieved and the pathway is activated, when IclR levels are low or when *iclR* is inactivated. This in particular occurs when cells are grown on acetate [36, 37], or in slow-growing glucose-utilizing cultures [38, 39].

The expression studies of *aceBAK* operon genes and *iclR* showed the repression of MPS phenotype by succinate in SM6 and SM11. ICL cleaves isocitrate to glyoxylate and succinate, and glyoxylate is oxidized by GO to produce oxalic acid [40]. Succinate in growth media induced the expression of *iclR*, which repressed *aceBAK* operon and oxalic acid-mediated MPS phenotype in SM6 and SM11. As *iclR* was not expressed in glucose-grown cells, the glyoxylate shunt and oxalic acid production were constitutive. Flow of carbon through the glyoxylate shunt can also be explained as a measure to utilize the acetate overproduced from pyruvate in glucose-grown cells. In *E*. *coli*, acetate production is usually credited to the “overflow metabolism” [40], and is believed to be a result of an imbalance between the energy requirement, biosynthesis and glucose uptake [41–43].

### *iclR* inactivation

In the *Enterobacteriaceae*, genes of the glyoxylate shunt are encoded in *aceBAK* operon and are under the direct negative control of repressor IclR [15, 16]. The expression of *aceBAK* is induced, when organisms are grown on acetate or fatty acids [44]. The glyoxylate bypass is required for growth on fatty acids and acetate, as they enter intermediary metabolism as acetyl-CoA. When *E*. *coli* is grown in excess of glucose, the carbon flux through glycolysis surpasses the capacity of the TCA cycle, and acetate accumulation occurs [45]. Difference in carbon flux through the glyoxylate shunt and the TCA cycle is observed in low acetate producer *E*. *coli* K strain (JM109) and high acetate producer *E*. *coli* B strain (BL21). In glucose-grown *E*. *coli* B cell, acetate is produced from pyruvate by pyruvate oxidase (PoxB). The acetate accumulated in cell can be converted to acetyl-CoA by acetyl-CoA synthetase (Acs), or by reversing the phosphotransacetylase-acetate kinase (Pta-AckA) pathway [46]. Acetyl-CoA is metabolized through the TCA cycle and the glyoxylate shunt by ICL and MS [44, 47]. Under these conditions, activation of the glyoxylate shunt in *E*. *coli* B strains (BL21) has been proposed as a route for internal acetate utilization (acetyl-CoA consumption), in contrast to *E*. *coli* K strain (JM109) where excess acetate is secreted out [48]. Acetyl-CoA utilization with a higher ICL activity by activation of the glyoxylate shunt has been observed in BL21 only and not in JM109 in high glucose batch cultures [48]. *K*. *pneumoniae* SM6 and SM11 apparently resembled *E*. *coli* B strain, where operation of the glyoxylate shunt is constitutive when glucose is sole carbon source. Moreover, absence of acetic acid production by SM6 and SM11 [14] also related them to *E*. *coli* B strain, in contrast to *E*. *coli* K strain.

### Physiological experiments and enzyme activities

The release of soluble P in the medium is the result of organic acid production [49]. The growth pattern of both *iclR* deletion strains was similar to that of parental wildtype strains, as reported earlier [14]. This showed that *iclR* inactivation did not interfere with glucose and succinate utilization. Unlike rhizobacteria *Pseudomonas* and *Rhizobium*, where C_4_ acids like succinate and malate are preferred over glucose, enterobacteria and firmicutes utilize glucose as the most preferred carbon source [50–52]. This is mainly because of the catabolite repression and carbon uptake PTS that controls the expression of carbon metabolism genes and carbon source uptake and subsequent utilization via EMP pathway. To further confirm that succinate mediated repression of MPS phenotype, activities of ICL and GO were analysed in glucose and succinate-grown cells of wildtype and SM6Δ and SM11Δ.

The ICL and GO activities measured in glucose- and succinate-grown SM6 and SM11 cells (Fig 3) were similar to those reported previously [14]. Interestingly, *iclR* inactivation did not alter ICL and GO activities of the glucose-grown cells. The possible explanation for these data is that the glucose-grown cells oxidise acetyl-CoA to glyoxylate via the glyoxylate cycle, and not via the classical TCA cycle, thus providing the substrate for oxalate synthesis.

### MPS phenotype of *iclR* mutants

Although the release of P mediated by *ΔiclR* strains in glucose+succinate media was still not as high as in the media containing glucose only, the results are encouraging and reveal that regulators of carbon metabolism influence the MPS phenotype, depending on the nature of an available carbon source. Derepression of the MPS phenotype during growth on glucose+succinate observed in *ΔiclR* strains was due to enhanced organic acid production. This effect was accompanied by the acidification and rock phosphate solubilization in TRP media. Thus, the carbon source-mediated repression of the MPS phenotype may be one of the reasons of failure of several phosphate solubilizing microorganisms to improve plant growth in the field experiments. Indeed, the availability of multiple carbon sources in natural conditions may lead to repression of various functional genes, including those responsible for MPS phenotype. Identification and inactivation of such regulators may abolish the repression, thus resulting in engineering of microbes to constitutive P solubilization.

### Plant experiments

Both, the wildtype and *ΔiclR* strains of *K*. *pneumoniae* SM6 and SM11 improved plant growth, whereas the mutant strains were significantly more efficient in terms of root and shoot length, root: shoot ratio and dry mass. This effect of *iclR* inactivation was not related to increase of IAA synthesis, but rather to the P release mediated by the oxalic acid production. Indeed, we showed that the synthesis of glyoxylate, oxalate precursor, was enhanced in the mutant. Thus, the repressor inactivation may result in better performance of PGP bacteria in the rhizosphere. Free-living N_2_ fixers with relieved constitutive MPS phenotype can serve dual purpose to plants.

## Supporting Information

S1 Fig Diagrammatic representation of pSMB5a and pSMB5b. (DOCX)

S2 Fig Strategy for insertional inactivation of *iclR* by allelic exchange. (DOCX)
