# Supplementary figures and images for: Derepression of Mineral Phosphate Solubilization Phenotype by Insertional Inactivation of iclR in Klebsiella pneumoniae
Source: PLoS One. 2015 Sep 18;10(9):e0138235. doi: 10.1371/journal.pone.0138235 (PMC4575152; doi:10.1371/journal.pone.0138235)

**S1 Fig:** Diagrammatic representation of pSMB5a and pSMB5b


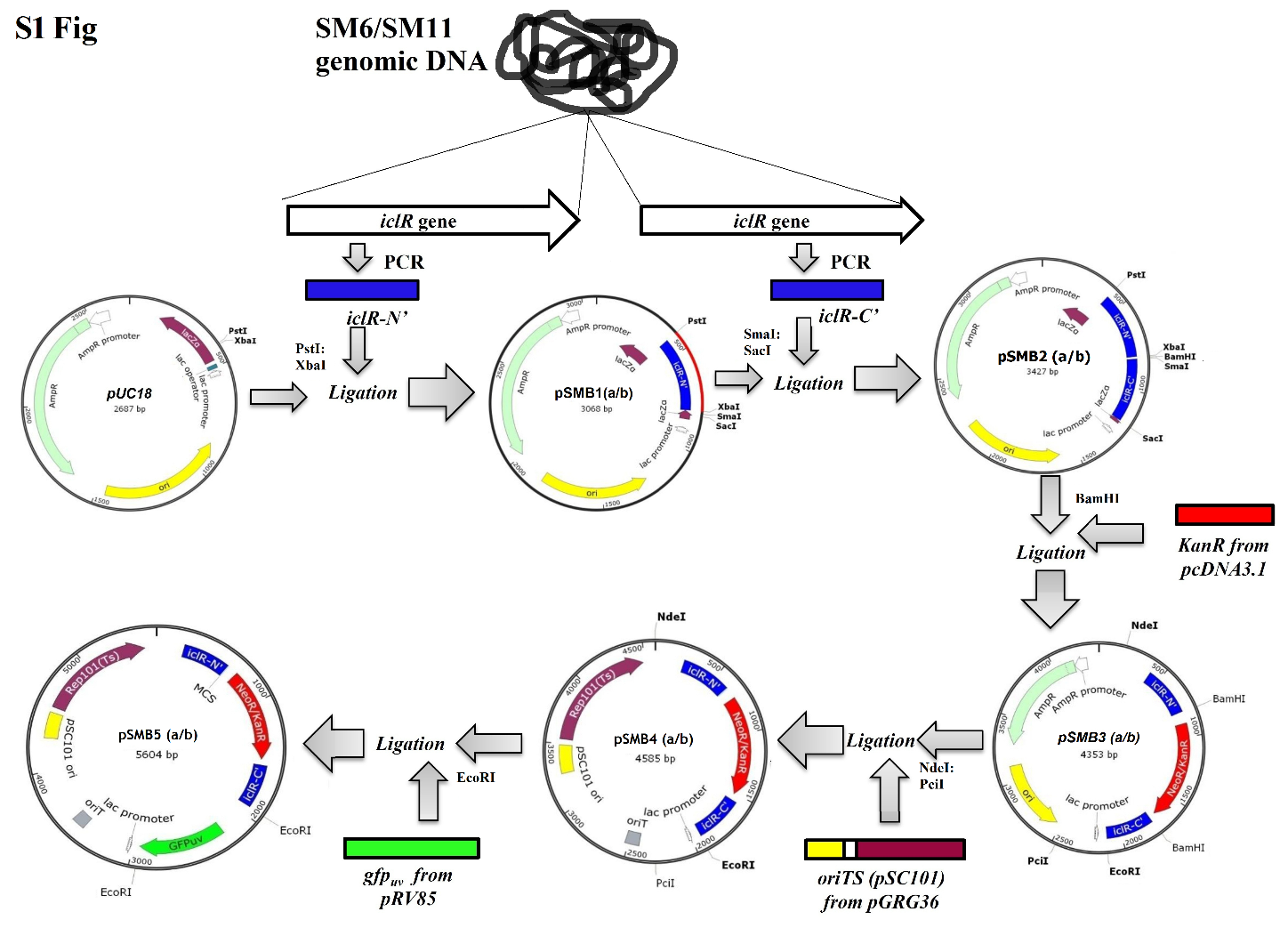

Supplement: S1 Fig — (DOCX) [file pone.0138235.s001.docx]

**S2 Fig:** Strategy for insertional inactivation of *iclR* by allelic exchange


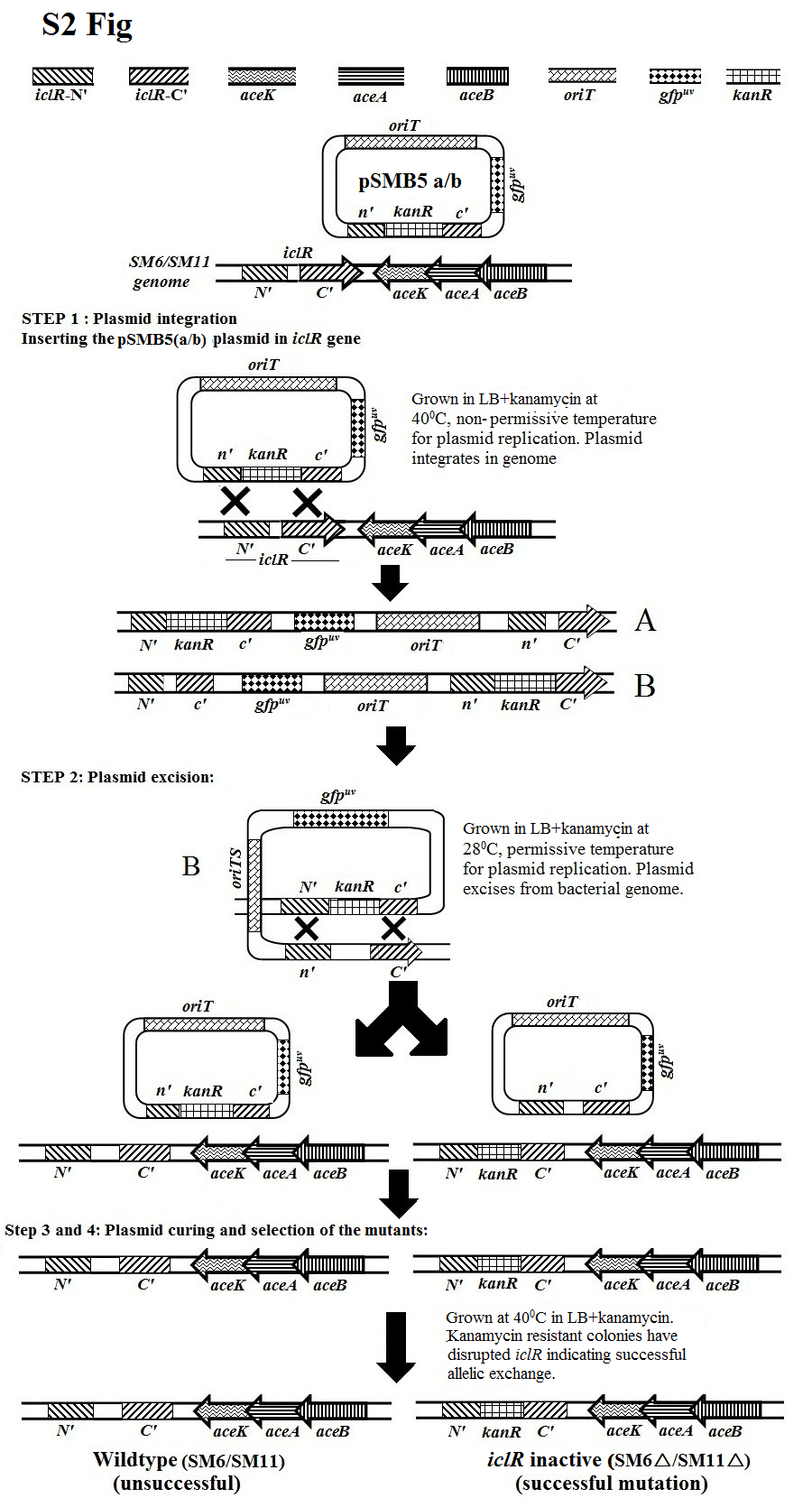

Supplement: S2 Fig — (DOCX) [file pone.0138235.s002.docx]
